# Changes in the Sprint, Vertical Jump and Quadriceps Strength after a Capacitive Resistive Electric Transfer Therapy Intervention—A Randomized Clinical Trial

**DOI:** 10.3390/sports12010036

**Published:** 2024-01-22

**Authors:** Max Canet-Vintró, Jacobo Rodríguez-Sanz, Carlos López-de-Celis, César Hidalgo-García, Guillermo R. Oviedo, Sergi Rodríguez-Rodríguez, Albert Pérez-Bellmunt

**Affiliations:** 1 Department of Basic Sciences, Faculty of Medicine and Health Sciences, Universitat International de Catalunya, 08195 Barcelona, Spain; maxcanet44@uic.es (M.C.-V.); goviedo@uic.es (G.R.O.); srodriguezr@uic.es (S.R.-R.); aperez@uic.cat (A.P.-B.); 2 ACTIUM Functional Anatomy Group, 08195 Barcelona, Spain; 3 Department of Physiotherapy, Faculty of Medicine and Health Sciences, Universitat International de Catalunya, 08195 Barcelona, Spain; 4 Fundació Institut Universitari per a la Recerca a l’Atenció Primària de Salut Jordi Gol i Gurina (IDIAPJGol), 08007 Barcelona, Spain; 5 Faculty of Health Sciences, University of Zaragoza, 50009 Zaragoza, Spain; hidalgo@unizar.es

**Keywords:** CRET therapy, sprint, muscle activity

## Abstract

Generating large mechanical power during actions such as sprinting or jumping is a crucial factor in many sports. These types of actions require a good warm-up activation. Capacitive-Resistive Electric Transfer (CRET) is a non-invasive therapy based on the application of radio frequency electric currents within the range of 300 kHz–1.2 MHz to accelerate tissue metabolic activity. This study aimed to evaluate the effectiveness of adding CRET to an active warm-up protocol in young adult athletes. For the double-blind randomized clinical trial, 60 healthy athletes were recruited and divided into an Experimental group (EG) and a Sham group (SG). EG received a CRET protocol in addition to an active warm-up. SG carried out the same warm-up but with a placebo CRET. The main outcome measures were isometric extension force, countermovement-jump (CMJ), 30 m-sprint test, and surface electromyography (sEMG). There is no statistically significant interaction (group–time) for any of the variables studied. Significant main effects for time were found in isometric extension force (*p* = 0.008); 30 m sprint (*p* = 0.017); rectus femoris sEMG during CMJ (*p* = 0.002); vastus lateralis sEMG during CMJ (*p* = 0.012); vastus medialis during CMJ (*p* = 0.010) and rectus femoris sEMG during the 30 m sprint test (*p* = 0.012). Non-significant differences between means are observed in the isometric extension force (48.91 EG; 10.87 SG) and 30 m sprint (−0.13 EG; −0.04 SG) variables. To conclude, a non-significant tendency was observed in sprint and quadriceps strength following CRET therapy, compared to the individuals’ pre-treatment state. Future research should use more treatment sessions to observe this tendency.

## 1. Introduction

The ability to generate a large mechanical power in the lower extremities during actions such as sprinting or jumping is a determinant factor in many sports [1,2], and muscle strength is also decisive in this type of sport performance [3]. The optimization of pre-competition activation is of paramount importance for athletes engaging in high-power activities. Preliminary warm-up routines exert a substantial influence on athletes’ performance during such competitions. Various priming activities, such as specific sprints and resistance training, have been shown to enhance performance on competition days, ensuring that athletes can fully utilize their training-derived improvements [4]. Different studies have analyzed independent sports actions and also, using various muscular activity-specific tools, have directly investigated the effects of different warm-up strategies in the performance of large-power actions, such as dynamic exercises that induce an activation after doing them, for improvements in sprinting and sustained large-intensity events [5,6].

A specific technique that measures muscular activity is surface electromyography (sEMG) [7,8]. sEMG is used to evaluate the electrical activity of muscles in response to nerve stimulation [9]. Muscle activity is often used to indicate muscle fatigue [10,11]. Anaerobic exercises lasting more than 10 s can provide information on muscle activity, fatigue onset, and performance decline. Other studies analyzing warm-up protocols relate good activation to an increased sEMG activity at the main muscle involved [9,12]. High levels of muscle activity in large-powered short actions are correlated with improved performance in these tests [12,13].

The literature agrees that active warm-up exercise protocols are typically used before starting a competition [5]. However, the most effective pre-competition warm-up protocol is not identified for the different types of sport [14,15,16]. Different passive strategies are also suggested to be combined with active warm-up protocols in order to improve performance further. Moist heat packs in contact with the muscle belly [15], pre-competition massage [17], or heating the area with a blizzard survival jacket before starting the competition [18] are examples of these passive classic strategies.

In the last ten years, new passive warm-up strategies have been developed [19,20,21]. One of these strategies is based on electrical or electromagnetic stimulation [15,16]. Electrical therapy has been combined with the use of radio frequency, resulting in Capacitive Resistive Electric Transfer (CRET) therapy, also known as Tecar therapy [22].

Capacitive Resistive Electric Transfer is a non-invasive therapy that applies electric currents within the radio frequency range of 300 kHz–1.2 MHz, which passes between an active and an inactive electrode [22,23,24]. The passage of electrical currents through the deep tissues of the body is associated with accelerated metabolic activity, an improvement of local circulation, and hemoglobin oxygenation of the tissues [24,25]. Previous cadaveric studies demonstrate the capacity of CRET therapy to reach deep structures such as the muscle or tendon [26,27]. Also, studies comparing CRET therapy versus sham treatments have recently been carried out in animals, specifically horses, obtaining the improvement of parameters such as power and running efficiency during trot velocities [23,24].

However, scientific evidence about the effects of CRET therapy on the performance of athletes is still lacking. Therefore, this study aimed to assess the efficacy of integrating CRET therapy into an active pre-competition warm-up protocol to enhance performances in maximal strength tests, vertical jumps, and 30 m sprints in young adult athletes.

## 2. Materials and Methods

### 2.1. Study Design

This study was a double-blind, randomized clinical trial carried out in August 2023. The study protocol was registered under ClinicalTrials.gov identifier NCT05892497 (accessed on 26 May 2023). The study protocol was approved by the local ethics committee (CBAS-2021-08) and complies with the principles of the Declaration of Helsinki [28]. Consolidated Standards of Reporting Trial (CONSORT) guidelines were followed throughout the study.

### 2.2. Participants

Participants were healthy athletes studying in the Faculty of Medicine and Health Sciences, who participated voluntarily after signing an informed consent. The participants were divided into two groups: both took part in an active warm-up; at the same time, the Experimental group (EG), received CRET therapy treatment, and the Sham group (SG) received the same treatment but in placebo form (with the machine switched off). Random assignment was performed using a computerized list randomizer (https://www.random.org/lists/, accessed on 3 March 2023), which generates a random list for the EG and SG.

We used the GRANMO 7.12 software to calculate the sample size, performing a two-sided test analysis and assuming an α risk of 0.05 and a β risk of 0.20. The common standard deviation and the minimum differences to be detected between the EG and SG were determined based on a pilot study with 15 participants. A common standard deviation of 0.89 and a minimum difference to be detected of 0.6 were used.

We established that a sample size of sixty participants (30 participants per group) was necessary. Inclusion criteria were: (a) healthy athletes whose sport involves the sprint task; (b) age between 18 and 35 years; and (c) to have signed the informed consent form. Exclusion criteria included: (a) volunteers who have suffered an injury during the last two months or were unable to perform physical activity; (b) persons who presented neurologic or orthopedic problems during the last year; (c) having received any lower limb surgical interventions during the last six months; (d) not understanding the orders provided by the researchers; and (e) participants who have reported allergies to conductive cream.

### 2.3. Variables

#### 2.3.1. Knee Extension Force

**Figure 1:**
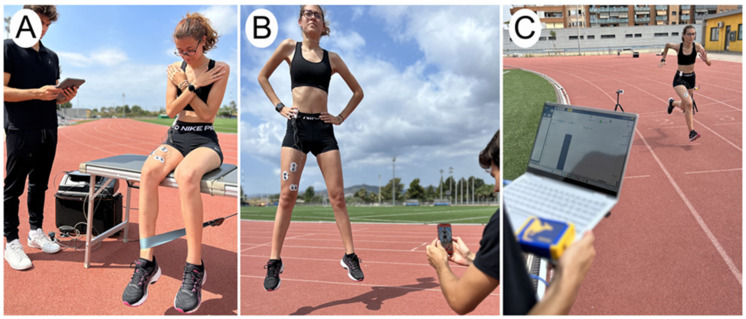
VARIABLES. (**A**) Knee extension force; (**B**) countermovement jump; (**C**) sprinting speed.

Quadriceps strength of the dominant lower limb was registered in Newtons. A traction dynamometer (PCE Ibérica S.L., Albacete, Spain) was used. This dynamometer has a 5% accuracy when measuring [29] and is highly reliable (ICC = 0.91; 95% CI = 0.76–0.97) [30]. During the assessment, participants were seated with their hips and knees flexed at a 90° angle, and a strap was secured on the distal and anterior part of the dominant leg. They were instructed to execute a knee extension, exerting maximal isometric force for 5 s. The test was repeated three times, and the mean value was used for the analysis (see Figure 1A).

#### 2.3.2. Vertical Jump Height

The assessment of vertical jumping ability utilized the My Jump 2 mobile phone application, which measures jump height (in cm) and propulsion force (in Newtons) during a Countermovement Jump (CMJ) with a high level of reliability (ICC = 0.813; 95% CI = 0.747–0.863) [31,32]. The CMJ involves the participant beginning in an upright position with knees fully extended, feet approximately shoulder-width apart, and hands on both iliac crests. Following the methodology outlined by Balsalobre et al. [33], participants aimed to achieve maximum height in their jump from this starting position and land in the same stance. My Jump 2 has demonstrated strong reliability and accuracy compared to the gold standard (force plate) [33]. Each participant performed three jumps, and the mean value was utilized for analysis (see Figure 1B).

#### 2.3.3. Sprinting Speed

The assessment of sprinting speed involved the utilization of the 30 m sprint test in conjunction with photocell sensors (Chronojump Boscosystem, Barcelona, Spain) [34,35]. These sensors, positioned at the starting line and 30 m apart, demonstrated exceptional reliability (ICC = 1.00; 95% CI = 1.000–1.00). Participants were instructed to achieve their maximum speed during the sprint, commencing from a static position and running to cross between the beams of both photocells. All sprint tests were conducted outdoors on an athletic track (see Figure 1C).

#### 2.3.4. Muscle Activity

Surface electromyography (sEMG) was used to evaluate the muscle activity of the quadriceps during the sprint, knee extension force and CMJ tasks. In a related study examining analogous variables (maximal isometric strength, vertical jump, sprint, and cutting), Fauth M. L. et al. [35] established the reliability of sEMG in quadriceps muscles. They discovered all ICC values to be above 0.80, with the majority exceeding 0.90. Furthermore, the validity of the sEMG mDurance^®^ system (mDurance Solutions SL, Granada, Spain) was confirmed for recording muscle activity during a functional task (ICC = 0.916; 95% CI = 0.831–0.958) [36]. The muscles assessed were vastus medialis, vastus lateralis, and rectus femoris. Data were obtained for the dominant limb.

The mDurance^®^ system (mDurance Solutions SL, Granada, Spain) consists of three parts: (a) a Shimmer3 sEMG unit (Realtime Technologies Ltd., Dublin, Ireland). This unit is a bipolar surface electromyography sensor for acquiring muscle activity. Each Shimmer3 has two channels, with a sampling rate of 1024 Hz. Shimmer3 applies a bandwidth of 8.4 Hz, and the sEMG signal resolution is 24 bits and has an overall amplification of 100 to 10,000 *v*/*v* [37]; (b) the mDurance Android application, which receives the data from the Shimmer3 and sends it to a cloud service [37]; (c) the mDurance cloud service where the data is stored, filtered, and analyzed [37]. For the processing and filtering of raw data, both isometric and dynamic tests were filtered using a fourth-order Butterworth bandpass filter with a 20–450 Hz cut-off frequency. The signal was smoothed using a window size of 0.025 s root mean square (RMS) and an overlapping of 0.0125 s between windows [37]. The Maximal Voluntary Isometric Contraction (MVIC) was calculated using the peak of the RMS signal during the extension knee isometric test. The RMS was the principal variable recorded for muscle activity, expressed as % of MVIC (%MVIC).

The participants’ skin was cleaned with alcohol and dried before the electrodes were placed. If hair impeded the correct adhesion of the electrodes to the skin, the particular site was shaved. Self-adhesive 5 × 5 cm Valutrode^®^ surface electrodes were placed on the muscle bellies according to the SENIAM project recommendations [38] and with an interelectrode distance of 20 mm [37]. Vastus medialis electrodes were placed at 80% on the line between the anterior superior iliac spine and the joint space in front of the anterior border of the medial collateral ligament of the knee, with an orientation almost perpendicular to this same line for the belly muscle. Vastus lateralis electrodes were placed between the line from the anterior superior iliac spine to the lateral side of the patella, and they were placed 2/3 s following the direction of the belly muscle. Finally, the electrodes for the recuts femoris were also placed on the midpoint between the anterior superior iliac spine and the patella midpoint for the belly muscle. Reference electrodes were placed at the patella midpoint and anterior superior iliac spine.

### 2.4. Intervention

A physical therapist took the test–retest measurements, and the intervention was carried out by another physical therapist familiar with CRET therapy treatments. Both therapists have over a decade of experience in physical therapy. The intervention was administered individually in the facilities of the Universidad International de Catalunya. Both groups of participants were given a single 30 min session.

The activation warm-up protocol for both groups comprised identical sets of active lower limb exercises. At the same time, the EG received the CRET therapy protocol with the tecartherapy machine (T-Plus Wintecare^®^). In the treatment of CRET therapy, the resistive modality and power doses specified below were used to achieve the passage of current to deep structures, increasing blood perfusion but without raising the temperature. So, the therapist informed the athletes that the treatment had no thermal effect, so their sensation should never be one of heat perception.

**Figure 2:**
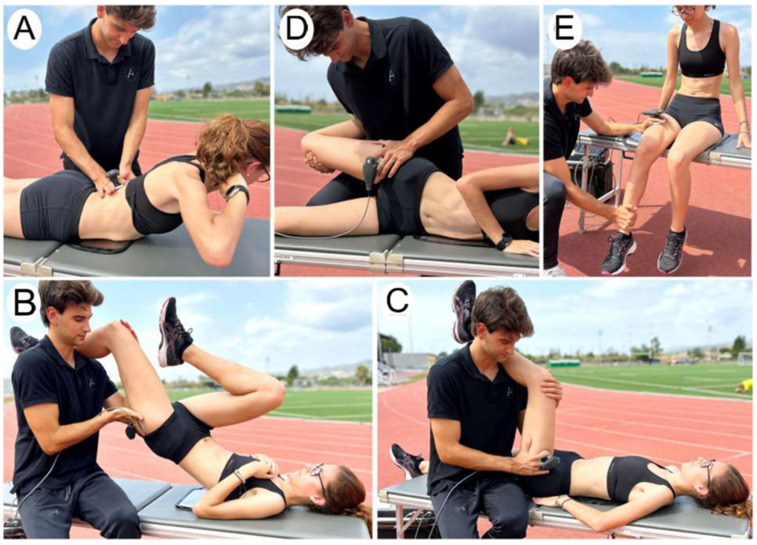
INTERVENTION. (**A**) Spinal extensors; (**B**) hip extensors; (**C**) hip flexors; (**D**) hip flexors; (**E**) quadriceps.

The activation protocol consisted of 5 exercises with five different powers applications. In the first exercise (see Figure 2A), the participants performed three sets of 8 repetitions of spinal extension while the therapist applied the CRET treatment in resistive mode at 30 W of power (see Figure 2A). The second exercise involved activating the hip extensor muscles by performing two sets of 8 repetitions with each leg of a unilateral gluteal bridge. Simultaneously, they explosively flexed the hip of their opposite leg, bringing the knee toward the chest. The therapist applied a dose of 30 W resistive CRET to the gluteal area. In the third exercise (see Figure 2C), the objective was a combination of two exercises to activate the hip flexors and knee extensors. In the first part of the exercise, the athletes were with the hip flexed at 90°, and the therapist resisted the hip flexion, causing an isometric contraction for 3-s, followed by 3 s of rest, which counted as one repetition. At the end of 8 repetitions, without a break, the therapist placed his knee under the participants’ knee, causing a 30°–45° knee flexion, and resisted the participants’ knee extension towards concentric contraction. During both exercises, the therapist applied a resistive dose of 40W to the insertion area of the rectus femoris first and then moved between the location of the vastus lateralis and medialis. Exercise 4 (see Figure 2D) consisted of activating the hip flexor musculature with two sets of 10 repetitions. The volunteers were side-lying, the leg on top was in a neutral position, and the therapist applied resistance to make a concentric contraction up to 90° of hip flexion. After that, the resistance of the therapist was towards eccentric contraction, returning to the initial position, and finishing one repetition. The therapist applied a resistive dose of 20 W on the psoas iliacus area. The fifth and last exercise (see Figure 2E) aimed to activate the quadriceps. The participants performed two sets of 10 repetitions of knee extensions in a seated position. The movement started with the knees flexed at 90° and finished in complete extension. The therapist performed a manual resistance, holding 2 s at the initial position followed by a gradual release of resistance until the complete extension of the knee. At the same time, a dose of 50 W was applied in a resistive mood.

On the other hand, the SG executed identical exercises following the same instructions, yet without the CRET therapy intervention. During this period, the equipment remained deactivated as part of a placebo treatment. To ensure proper blinding, neither group could view the equipment screen. Furthermore, as the therapy was non-thermal, participants did not feel any increase in temperature.

### 2.5. Statistical Analysis

Statistical analysis was conducted with the SPSS 23.0 package (IBM, Armonk, NY, USA). There was no loss of follow-up in the study. The mean, standard deviation, F value and differences between means and 95% confidence interval were calculated for each variable. For the comparative analysis, the two-way ANOVA 2 × 2 (Group × Time) test was used. In the case of finding interaction, the 2 × 2 analysis was performed with Bonferroni correction.

Effect sizes (ES) were calculated using partial eta squared (ŋ^2^). Considering an effect size > 0.140 as large, around 0.060 are medium, and <0.039 small [39]. The data of all recruited participants were included in the final analysis. The level of significance was set at *p* < 0.05.

## 3. Results

**Figure 3:**
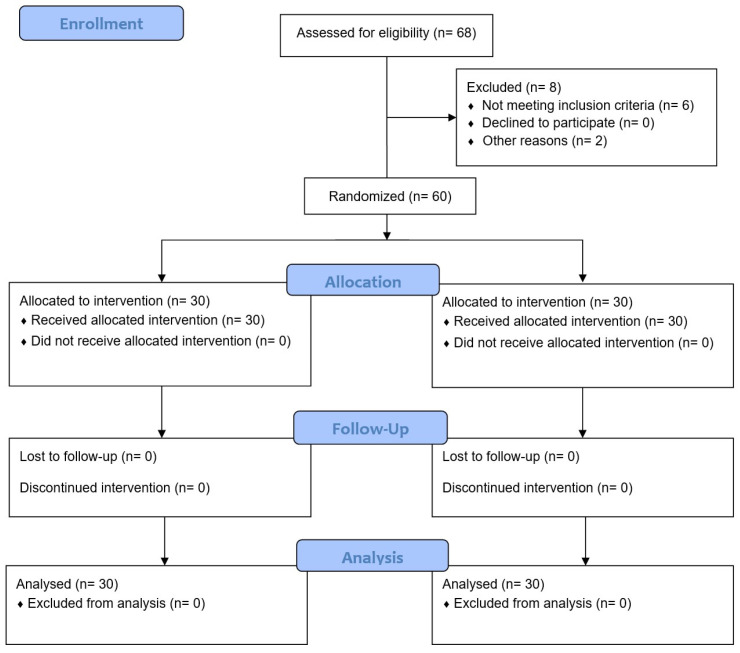
CONSORT. (Consolidated Standards of Reporting Trial) flow diagram.

In August 2023, 60 participants (EG, n = 30; SG, n = 30) were recruited. All participants met all eligibility criteria and agreed to participate. Then, the participants were randomly assigned to each group and received their assigned treatment. Enrolment and exclusions after randomization can be seen in Figure 3. The demographic characteristics of the sample are summarized in Table 1. No adverse events or side effects were reported for any participant.

There is no statistically significant interaction (group–time) for any of the variables studied (Table 2 and Table 3).

There were significant main effects in isometric extension force for time (F = 7.471; *p* = 0.008; ŋ^2^ = 0.11) but not for group (F = 3.026; *p* = 0.316; ŋ^2^ = 0.17).

In CMJ variable there were no significant main effects for either time (F = 0.514; *p* = 0.476; ŋ^2^ = 0.01) or group (F = 2.431; *p* = 0.124; ŋ^2^ = 0.04).

In the case of the 30 m sprint variable, there were significant main effects for time (F = 5.984; *p* = 0.017; ŋ^2^ = 0.09) but not for group (F = 3.123; *p* = 0.082; ŋ^2^ = 0.05).

In the case of sEMG during isometric extension force, there were no significant main effects for either time (F = 0.297; *p* = 0.588; ŋ^2^ = 0.01) or group (F = 0.123; *p* = 0.727; ŋ^2^ = 0.00) in rectus femoris activation. There were also no significant main effects for time (F = 0.677; *p* = 0.414; ŋ^2^ = 0.01) or group (F = 0.075; *p* = 0.785; ŋ^2^ = 0.00) in vastus lateralis activation. Finally, there were also no significant main effects for time (F = 0.255; *p* = 0.616; ŋ^2^ = 0.00) or group (F = 0.001; *p* = 0.990; ŋ^2^ = 0.00) in vastus medialis activation (Table 3).

In relation to the activation of sEMG during CMJ, there were significant main effects for time (F = 10.798; *p* = 0.002; ŋ^2^ = 0.16) but not for group (F = 0.421; *p* = 0.519; ŋ^2^ = 0.01) in rectus femoris activation. There were also significant main effects for time (F = 6.744; *p* = 0.012; ŋ^2^ = 0.11) but not for group (F = 0.144; *p* = 0.706; ŋ^2^ = 0.00) in vastus lateralis activation. Finally, there were also significant main effects for time (F = 7.020; *p* = 0.010; ŋ^2^ = 0.11) but not for group (F = 0.021; *p* = 0.886; ŋ^2^ = 0.00) in vastus medialis activation (Table 3).

In the sEMG during the sprint, there were significant main effects for time (F = 6.683; *p* = 0.012; ŋ^2^ = 0.10) but not for group (F = 2.035; *p* = 0.159; ŋ^2^ = 0.03) in rectus femoris activation. There were no significant main effects for time (F = 3.792; *p* = 0.056; ŋ^2^ = 0.06) or group (F = 0.108; *p* = 0.743; ŋ^2^ = 0.00) in vastus lateralis activation. Finally, there were no significant main effects for time (F = 1.791; *p* = 0.186; ŋ^2^ = 0.03) or group (F = 1.341; *p* = 0.252; ŋ^2^ = 0.02) in vastus medialis activation (Table 3).

## 4. Discussion

This study aimed to assess the potential efficacy of incorporating CRET therapy into an active pre-competitive warm-up protocol, specifically aiming to enhance performances in maximal strength tests, vertical jumps, and 30 m sprints.

According to the initial objective of this study, no statistically significant differences (group–time interaction) were observed in any variable. However, there is a non-significant tendency of superior improvement of the EG with respect to the SG in the variable’s isometric extension force, CMJ and 30 m sprint. If we observe the differences between means in Table 2, the EG improves isometric extension force almost 5 times more than the SG and 3 times more than the sham group in the 30 m sprint. Despite these results, no statistically significant changes were observed.

The sEMG results of both groups, no significant differences were observed in muscle activation during isometric extension force, CMJ and 30 m sprint test.

One of the most remarkable findings of the current clinical trial was the non-significant improvement of 0.13 s in 30 m sprint time in the EG. Several studies have suggested that neuromuscular and biomechanical factors may also be important determinants of running performance [40]. Duñabeitia et al. [22] have found improvements in runners produced by CRET therapy in neuromuscular coordination and spatiotemporal biomechanical factors, which are directly related to enhanced performance in athletes.

During an activation warm-up, the sympathetic nervous system releases catecholamines, which play a major role in the utilization of substrates by the skeletal muscle [41]. Catecholamines do not contribute directly to skeletal muscle contraction; however, they do enhance skeletal muscle contraction after prolonged rapid stimulation of motor neurons. This effect is believed to stem from the activation of presynaptic α-1 adrenergic receptors (ARs) located on motor nerve terminals [42,43]. During high-intensity activities like sprinting, the release of catecholamines triggers the mobilization of energy substrates, such as phosphocreatine, to support the increased energy demands of the muscles [44]. Hence, the potential impact of CRET therapy on increasing blood perfusion in the muscle may be associated with the heightened delivery and acceleration of catecholamines to the muscle [24,25], subsequently influencing the levels of phosphagen substrate, thereby contributing to enhanced sprint performance. In addition, the application of CRET therapy also produces a slight increase in temperature (around 2.37 °C to 5 °C higher than the temperature before receiving the treatment), which is also related to these physiological changes that positively affect the sprint performance [27].

The increase in knee extension strength observed in the EG (48.91 N) may be attributed to physiological changes in the muscle tissue induced by the electric current flow associated with CRET therapy. This effect is supported by the findings of various studies, which suggest that CRET intervention has the potential to improve muscle strength and performance, particularly in activities such as jumping and sprinting [24,25]. However, it is important to note that while these results are promising, further research is needed to fully understand the implications of CRET therapy on dynamic muscle performance.

In the vertical jump height test, the changes are smaller than 1 cm in both groups. These results may be due to CMJ is a dynamic test of a shorter duration than the 30 m sprint test, detecting differences in the CMJ may require more treatment sessions. Changes in performance in dynamic tests after CRET therapy, whether in humans or animals, were reported after two [22,23] and four sessions [24] of treatment.

Contrary to the initial hypothesis, no interaction differences were obtained in any of the studied variables. These results are related to the fact that our treatment with CRET therapy was only once. In contrast, the previous bibliography [22,23,24], which did obtain differences, implemented more sessions.

The findings of our study suggest that the incorporation of CRET therapy into a warm-up routine involving active muscle contractions not only enhances the baseline physiological condition of the muscles before a 30 m sprint test but also leads to a non-significant improvement in muscle strength compared to the athlete’s baseline state prior to the warm-up and therapy. These results may underscore the potential of CRET intervention to positively impact muscle function and performance, particularly in the context of activities such as sprinting.

This study has several limitations. Because the study was conducted on a day other than competition, the results may not necessarily be applicable to real-life athletic situations. In addition, since the participants in the study played a variety of sports and spent varying amounts of time practicing each week, it may be difficult to generalize the results to a specific sport or training regimen. Another limitation of the study is that there is no comparison with other therapies to see which therapy has greater thermal effects.

As future lines of research, it could be interesting to propose studies aimed to explore the effects of a more prolonged protocol, consisting of multiple sessions over several days, on athletes’ sprinting performance and evaluate its effects in different sprint distances (i.e., 60 m, 100 m or 200 m), and to compare the impact of CRET therapy with other hyperthermia therapies. The tendency observed in this study is interesting and it is possible that the results could be significant with the application of more treatment sessions.

## 5. Conclusions

This is the first study implementing CRET therapy during an active pre-competition warm-up protocol in young adult athletes. Our results suggest a non-significant enhancement in sprint performance and quadriceps strength following CRET therapy, when combined with muscle active contraction, compared to the individuals’ pre-treatment state. However, no discernible differences were observed in the group–time interaction, indicating that the efficacy of CRET therapy in improving performance remains uncertain. Future research should use more treatment sessions to assess whether the observed tendency reaches statistical significance. Additionally, the application of CRET therapy in sports and rehabilitation settings warrants further investigation to elucidate its effects, particularly in individuals from different sports and in diverse clinical conditions.

## Figures and Tables

**Table 1:** Demographic characteristics of the sample.

	Experimental Group (n = 30)	Sham Group (n = 30)
Age (years)	20.6 ± 2.96	20.7 ± 2.64
Sex	63.3% Men 36.7% Women	60% Men 40% Women
Dominance	80% Right 20% Left	83.3% Right 16.7% Left
Height (cm)	173.5 ± 8.65	173.7 ± 9.75
Weight (kg)	65.2 ± 12.12	69.2 ± 13.9
Hours of sport per week	7.5 ± 2.4	7.7 ± 3.0

**Table 2:** Comparison of isometric extension force, countermovement vertical jump, and 30 m sprint test.

Outcome/Group	Pre-Treatment	Post-Treatment	Mean Difference (95% CI)	Group–Time Interaction
Isometric Extension Force (Newtons)				
Experimental Group	327.30 ± 110.75	376.21 ± 124.55	48.91 (17.96, 79.87)	*p* = 0.087 F = 3.026 ŋ^2^ = 0.50
Sham Group	379.06 ± 148.64	389.93 ± 141.77	10.87 (−20.09, 41.82)	
Countermovement vertical jump CMJ (cm)				
Experimental Group	34.71 ± 7.99	35.18 ± 7.65	0.47 (−0.90, 1.84)	*p* = 0.799 F = 0.065 ŋ^2^ = 0.01
Sham Group	31.60 ± 8.23	31.82 ± 9.08	0.22 (−1.15, 1.60)	
30 m Sprint (s)				
Experimental Group	4.83 ± 0.44	4.70 ± 0.42	−0.13 (−0.22, −0.03)	*p* = 0.270 F = 1.238 ŋ^2^ = 0.21
Sham Group	5.05 ± 0.72	5.01 ± 0.72	−0.04 (−0.14, 0.05)	

Abbreviations: CMJ: countermovement vertical jump, CI: confidence interval.

**Table 3:** Comparison of electromyography values in the rectus femoris, vastus lateralis, and vastus medialis muscles during the countermovement vertical jump test, isometric extension force and 30 m sprint test.

Outcome/Group	Pre-Treatment	Post-Treatment	Mean Difference	Group–Time Interaction
Rectus Femoris RMS in CMJ (%)				
Experimental Group	63.50 ± 34.74	45.77 ± 19.99	−17.73 (−34.08, −1.37)	*p* = 0.828 F = 0.048 ŋ^2^ = 0.01
Sham Group	69.77 ± 57.45	49.52 ± 25.92	−20.25 (−36.60, −3.89)	
Vastus Lateralis RMS in CMJ (%)				
Experimental Group	69.78 ± 37.15	64.78 ± 27.11	−5.00 (−17.15, 7.16)	*p* = 0.155 F = 2.081 ŋ^2^ = 0.35
Sham Group	73.63 ± 28.06	56.96 ± 22.98	−16.67 (−29.84, −5.12)	
Vastus Medialis RMS in CMJ (%)				
Experimental Group	75.68 ± 61.71	53.66 ± 19.18	−22.02 (−39.17, −4.88)	*p* = 0.327 F = 0.975 ŋ^2^ = 0.17
Sham Group	68.63 ± 26.11	58.57 ± 25.93	−10.06 (−27.20, 7.08)	
Rectus Femoris RMS in isometric extension force (%)				
Experimental Group	46.16 ± 12.67	43.59 ± 13.36	−2.57 (−8.28, 3.16)	*p* = 0.473 F = 0.522 ŋ^2^ = 0.09
Sham Group	43.72 ± 13.98	44.08 ± 12.98	0.36 (−5.36, 6.08)	
Vastus Lateralis RMS in isometric extension force (%)				
Experimental Group	44.05 ± 11.00	42.37 ± 12.99	−1.68 (−7.72, 4.36)	*p* = 0.973 F = 0.001 ŋ^2^ = 0.00
Sham Group	44.86 ± 13.68	43.03 ± 15.11	−1.83 (−7.87, 4.21)	
Vastus Medialis RMS in isometric extension force (%)				
Experimental Group	44.82 ± 12.64	41.36 ± 13.36	−3.46 (−9.43, 2.49)	*p* = 0.258 F = 1.304 ŋ^2^ = 0.22
Sham Group	42.39 ± 13.18	43.73 ± 14.07	1.34 (−4.62, 7.30)	
Rectus Femoris RMS in Sprint (%)				
Experimental Group	240.79 ± 209.84	148.92 ± 178.78	−91.87 (−168.26, −15.48)	*p* = 0.416 F = 0.671 ŋ^2^ = 0.11
Sham Group	172.60 ± 138.01	124.94 ± 106.21	−47.66 (−124.05, 28.73)	
Vastus Lateralis RMS in Sprint (%)				
Experimental Group	214.53 ± 216.86	404.28 ± 574.84	379.75 (−76.30, 455.80)	*p* = 0.943 F = 0.005 ŋ^2^ = 0.00
Sham Group	190.56 ± 206.65	366.86 ± 794.78	176.30 (−89.75, 442.35)	
Vastus Medialis RMS in Sprint (%)				
Experimental Group	268.71 ± 521.13	565.64 ± 763.96	296.93 (−2.15, 596.00)	*p* = 0.146 F = 2.167 ŋ^2^ = 0.36
Sham Group	278.10 ± 789.26	263.96 ± 383.51	−14.14 (−313.21, 284.94)	

Abbreviations: CMJ: countermovement vertical jump, CI: confidence interval.

## Data Availability

The data presented in this study are available on request from the corresponding author.
